# Is Scarlet Fever a Septicemia?

**Published:** 1905-06

**Authors:** L. B. Clark


ATLANTA

### Journal-Record of Medicine.

Successor to Atlanta Medical and Surgical Journal, Established 1855, and Southern Medical Record, Established 1870.

OWNED BY THE ATLANTA MEDICAL JOURNAL CO.

Published Monthly.

VOL. VII. JUNE, 1905. No. 3.

BERNARD WOLFF, M.D., EDITOR, Nos. 319-20 Prudential.

M. B. HUTCHINS, M.D., BUSINESS MANAGER, 1007-1008 Century Bldg.

J. N. LECONTE, M.D., ASSOCIATE EDITOR.

### ORIGINAL COMMUNICATIONS.

### IS SCARLET FEVER A SEPTICEMIA?

BY L. B. CLARKE, M.D.

The intent and purpose of this article is not in the light of an attempt to place any stumbling-block in the way of or to otherwise impede the scientific search for the hitherto unknown primary factor producing scarlet fever in my own mind (I do not expect to influence others), but, as the question-mark following the title of this paper indicates, it is a desire to evolve an open discussion of the peculiar manifestations of this disease, a comparison of the deductions drawn from the writer's observations in dealing with the malady, with the inferences of others who have come in contact with it, and a study of the complete septic picture clinically presented in a well-marked case, with the possible resultant conclusion that (1) there is probably no factor in the production of the disease other than is now known; (2) the disease, in its manifestations, is certainly a simon-pure streptococcus poisoning—sepsis.

It is passing strange, that with the advanced methods and opportunities of science to-day for original investigation, and the great array of specific organisms that have been found for us, one producing scarlet fever has not yet been isolated. Why is this search unsuccessful? Is it because none exists, or is it due to incapacity on the part of the present microscope?

It is true that we are in the same darkness concerning many other diseases—syphilis, cancer, and nearly all of the highly contagious diseases; but in these we have no such known factor as the streptococcus, which we have positively had time and again demonstrated to exist in scarlet fever, having been found in the blood, and post-mortem in the skin, glands and kidneys.

The streptococcus manifests its presence in scarlet fever by angina, otitis media, endocarditis, arthritis. Nearly all of the serious complications in the disease may justly be accredited to it, even, according to some authority, nephritis and pneumonia.

Baginsky found the organism in the heart's blood and bone marrow in almost every fatal case, and in the body in 696 out of 701 cases examined.

It is the writer's opinion that nephritis is caused by the extensive destruction of the skin; burns and scalds have the same tendency.

To trace from beginning to end the clinical manifestations of a well-marked case of scarlet fever, including some, at least, of the complications that occur in such cases, the fairly close observer will be struck with the similarity of the disease to the cases of septic infection he has had under care; not alone in the general array of symptoms presented, nor in the choice of structures selected for involvement, but in the excellent results obtained from practically the same treatment arising from the identical indications.

From an etiological standpoint, if it were conclusively proven that the disease is entirely communicated through the respiratory tract, the ideas presented in this paper could have no tenable ground; but, such is not the case, inasmuch as we have undoubted record of epidemics arising from the carrying of the disease into the alimentary canal by infected milk, thus giving evidence that the essential initial point of invasion is not necessarily the pharynx.

It appears, therefore, knowing additional vices of the streptococcus, that if we are to assume that the introduction into the blood of an individual of a streptococcus infection at times causes scarlet fever, we must admit that there exists *certain required conditions* under which only this may take place; and if so, what may be these *certain required conditions?*

There are those who would reply that, first, these certain required conditions are the presence of the specific organism; second, that streptococcus infection may play one of several roles in the animal economy; not necessarily scarlet fever.

We will first take up the latter. That is just the point.

Disease is very regular in one characteristic—its irregularity. No two individuals are affected alike in any infection; nor have they the identical sets of symptoms or complications. The same introduction of any disease, from the same source, into two or more individuals, is nearly sure to produce not alone dissimilar, but in the majority of instances, vastly different clinical pictures, particularly with regard to what apparently chance complications may ensue, due in the main to the course it may suit the infection to travel after entrance.

We know this to be especially true of all forms of blood poison, and where is the peculiarity more distinctly demonstrated than in syphilis? Why does it expend its fury on one sufferer in the various skin lesions, on another in gummata. Why does one have a gumma in the brain, another in the liver, another bone disease, etc. Again, tuberculous midwives have communicated the disease to infants delivered by them, which assumed the form of tuberculous meningitis. Why not some other tubercular manifestation? So much for general characters.

Now, let us analyze the etiological question contained in the first provision, which calls for *certain required conditions,* and which would ordinarily be allotted to the specific organism.

Let us note some of the varied forms of disease resulting from the different methods of entrance into the body of some other pathogenic organisms.

The tubercle bacillus plays its role in the economy dependent upon its mode of entrance, generally—

Entering by the respiratory tract it attacks lungs and bronchial glands, and our result is tuberculosis of the lungs.

Enter by the skin route, and we have lupus.

Enter by the blood, either primarily or secondarily, and the result is acute tuberculosis.

A more extensive example is anthrax. The varied effects of the bacilli and the spores from different modes of entrance into the body is much more remarkable than would be the same reasoning applied to the streptococcus.

*Animals inhale* anthrax spores, and die of *typical anthrax*. The spores establish in the lungs, penetrate the epithelium, and entering the blood produce the disease.

*Inhale the bacillus*; there ensues rapid multiplication. No sporulation and a violent *irritative pneumonia*, but no *general infection*.

The *bacilli in the stomach* are *killed* by the gastric juice.

The *spores in stomach* withstand the gastric juice, pass into intestines, develop into bacilli, and produce *general infection*.

Lastly, bacilli entering through skin lesions result in *malignant pustule*.

Would it not appear, therefore, that the foregoing examples of the results of varied invasion of the same organism, could with impunity be expected of other organisms?

Can we not venture the deduction that the *certain required conditions*, under which the streptococcus may produce scarlet fever, are not the presence of a specific organism, but the mode of entrance of these bacteria into the body?

May we not expect the pathologist of the future to succeed in demonstrating that its entrance through the respiratory and alimentary tracts, produce scarlet fever? All other methods of entrance, wounds, etc., result in one of the other earious forms of the same disease—blood poison.

### PATHOLOGY.

There is no morbid anatomy peculiar to scarlet fever. The rash disappears after death, and it is interesting to note in this connection these peculiar facts:

That the so-called *specific* infectious diseases, in which no characteristic visceral lesions have been found, are those in which the specific poison has not been found, as in scarlet fever, measles, German measles, chicken-pox, whooping-cough, while on the other hand, those found to possess definite characteristic visceral lesions are those in which the specific poison is a known definite quality, as typhoid fever, diphtheria, tuberculosis.

There are a few irregular exceptions to this condition :

Smallpox, mumps, but of the latter little is known ; there are no opportunities to study the gland pathologically.

Is there any significance attachable to this absence of pathology?

Under the symptomatology it is the writer's intention to deal generally, and with only those of interest to us in this argument, by their specially strong similarity to the symptoms of blood poison, and those complications presenting the same characteristics.

Indeed the complications are identical, if they can be called complications, except as they may become separate, independent pathological conditions, requiring independent procedures.

The general aspect of a severe infection is that of a severe septicemia from any other cause. The temperature is high, and is almost steady, fluctuation for many days, very little noted. The marked restlessness, dry, leathery tongue, sordes, complete anorexia; difficulty with which both food and medicine are given. Rapid, weak, irregular pulse. The typhoid condition. Delirium. The swelling of the glands of the neck ; the cellulites, are the same as would occur from an infected wound in that region. The tendency to abscess and slough, involvement of the lymph, nodes. Otitis media, mastoid abscess suppuration, the terrific prostration upon cessation of its process all present a complete septic picture, and point to a destructive agency in the blood ; a septic element, in many cases leading to the formation of abscesses in many parts, and a few going on to a definite pyemia.

Surgical scarlet fever would appear to bring additional strength to the value of this idea. Surgical patients are known to be peculiarly susceptible to scarlet fever, and it is serious. The patient dies with the general symptoms of septicemia.

There may or not be throat symptoms and may be no rash. Why does this susceptibility of surgical cases to scarlet fever infection, above all other exanthemas, exist, even in instances in which the surgery has been thoroughly and aseptically performed, and the operation of a most minor nature?

How much difference is there in the rational treatment of a case of scarlet fever of severe poisoning, and a septicemia from puerperal infection, wound infection or what not.

I believe alcohol to be the sheet-anchor in both.

Tinct. chlor. iron is largely used in both.

It is my own practice to utilize both of these drugs. The same cardiac stimulants are needed in both. The same disinfection of any visible area of infection, be it in the throat of the one, or lesion elsewhere in the other.

The same ichthyol to abort abscess formation, and if it fail the same surgical procedures to evacuate pus, to prevent its absorption.

About two years ago it fell to my lot to uphold the medical end of an attack of septicemia, brought about in the person of one of our confreres by having infected himself in operating upon an empyema. Associated in the care of our brother were two of our prominent surgeons. About the time he recovered, I had under care a child of five years with a severe scarlet fever.

The chief difference between the medical treatment of the two consisted in a reduction of dose to suit the age of the child.

